# The preoperative triglyceride-glucose index has a positive effect on predicting the risk of short-term restenosis after carotid artery stenting: a retrospective cohort study

**DOI:** 10.3389/fneur.2023.1159601

**Published:** 2023-04-17

**Authors:** Shan-shan Zhao, Zhen-zhen Jiang, Bo Wei, Jian-bo Zhu, Xia-tian Liu

**Affiliations:** ^1^Department of Ultrasound, Shaoxing People’s Hospital, Shaoxing, China; ^2^Department of Neurology, Shaoxing People’s Hospital, Shaoxing, China

**Keywords:** TyG index, insulin resistance, carotid artery stenting, in-stent restenosis, adverse event

## Abstract

**Background:**

Increasing evidence suggests that insulin resistance is linked to cardiovascular disease and atherosclerosis. The triglyceride–glucose (TyG) index has proven to be a convincing marker to quantitatively evaluate insulin resistance. However, there is no relevant information about the relationship between the TyG index and restenosis after carotid artery stenting.

**Methods:**

A total of 218 patients were enrolled. Carotid ultrasound and computed tomography angiography were used to evaluate in-stent restenosis. A Kaplan–Meier analysis and Cox regression method were performed to analyze the correlation between TyG index and restenosis. Schoenfeld residuals were used to determine the proportional-hazards assumption. A restricted cubic spline method was used to model and visualize the dose–response relationship between the TyG index and the risk of in-stent restenosis. Subgroup analysis was also performed.

**Results:**

Thirty-one participants (14.2%) developed restenosis. The preoperative TyG index had a time-varying effect on restenosis. Within 29 months post-surgery, an increasing preoperative TyG index was linked to a significant increased risk of restenosis (hazard ratio: 4.347; 95% confidence interval 1.886–10.023). However, after 29 months, the effect was decreased, although not statistically significant. The subgroup analysis showed that the hazard ratios tended to be higher in the age ≤ 71 years subgroup (*p* < 0.001) and participants with hypertension (*p* < 0.001).

**Conclusion:**

The preoperative TyG index was significantly associated with the risk of short-term restenosis after CAS within 29 months post-surgery. The TyG index may be employed to stratify patients based on their risk of restenosis after carotid artery stenting.

## 1. Introduction

A global investigation published in 2014 showed that an approximated 6 million people died from stroke worldwide in 2013, with ischemic stroke accounting for 51% of all deaths (1). Severe internal carotid artery (ICA) stenosis is associated with a higher risk of ischemic stroke, and carotid artery stenting (CAS) is a popular minimally invasive treatment option for carotid stenosis. Moreover, it has been demonstrated that CAS is comparable to carotid endarterectomy in terms of lowering the risk of stroke (2). In-stent restenosis (ISR) is a major complication that affects long-term safety and efficacy. The incidence of restenosis after CAS is reported to be around 5–20% (3, 4). Although restenosis is mostly asymptomatic, it may require a second intervention, and this increases the risk of ipsilateral ischemic stroke. Therefore, early identification of convenient and accurate biomarkers of ISR is critical for patients with CAS.

Increasing evidence suggests that insulin resistance (IR) is linked to cardiovascular disease and atherosclerosis (5, 6). A previous study also reported that IR is related to restenosis after coronary stenting (7). The homeostasis model assessment index was applied in this study to assess IR. However, although this index is considered the gold standard, it is expensive and clinically inconvenient. IR can lead to hyperglycemia and increased serum triglyceride (TG) levels. On this basis, the TyG index, calculated from serum fasting blood glucose (FBG) and TG, has proven to be a convincing indicator for quantitative evaluation of IR (8). Recent research has demonstrated that a higher TyG index increases the risk of ISR after coronary stenting (9). However, no relevant information regarding the effect of the TyG index on restenosis after CAS is known. Moreover, our previous research (10) showed that a higher TyG index can increase the burden of carotid plaque in patients with new-onset diabetes. Accordingly, we hypothesized that the TyG index may also contribute to restenosis after CAS. Therefore, the aim of our study was to investigate the relevance of the preoperative TyG index on the risk of ISR in participants who underwent CAS for carotid atherosclerotic stenosis. The results of our study may assist in the identification of individuals at high risk of restenosis after CAS.

## 2. Methods

### 2.1. Ethics statement

This study was reviewed and approved by the Academic Ethics Committee of Shaoxing People’s Hospital (ID: 2020-K-Y-070-01). Written informed consent for participation was not required for this study in accordance with the National Legislation and the Institutional Requirements.

### 2.2. Study design

**Figure 1:**
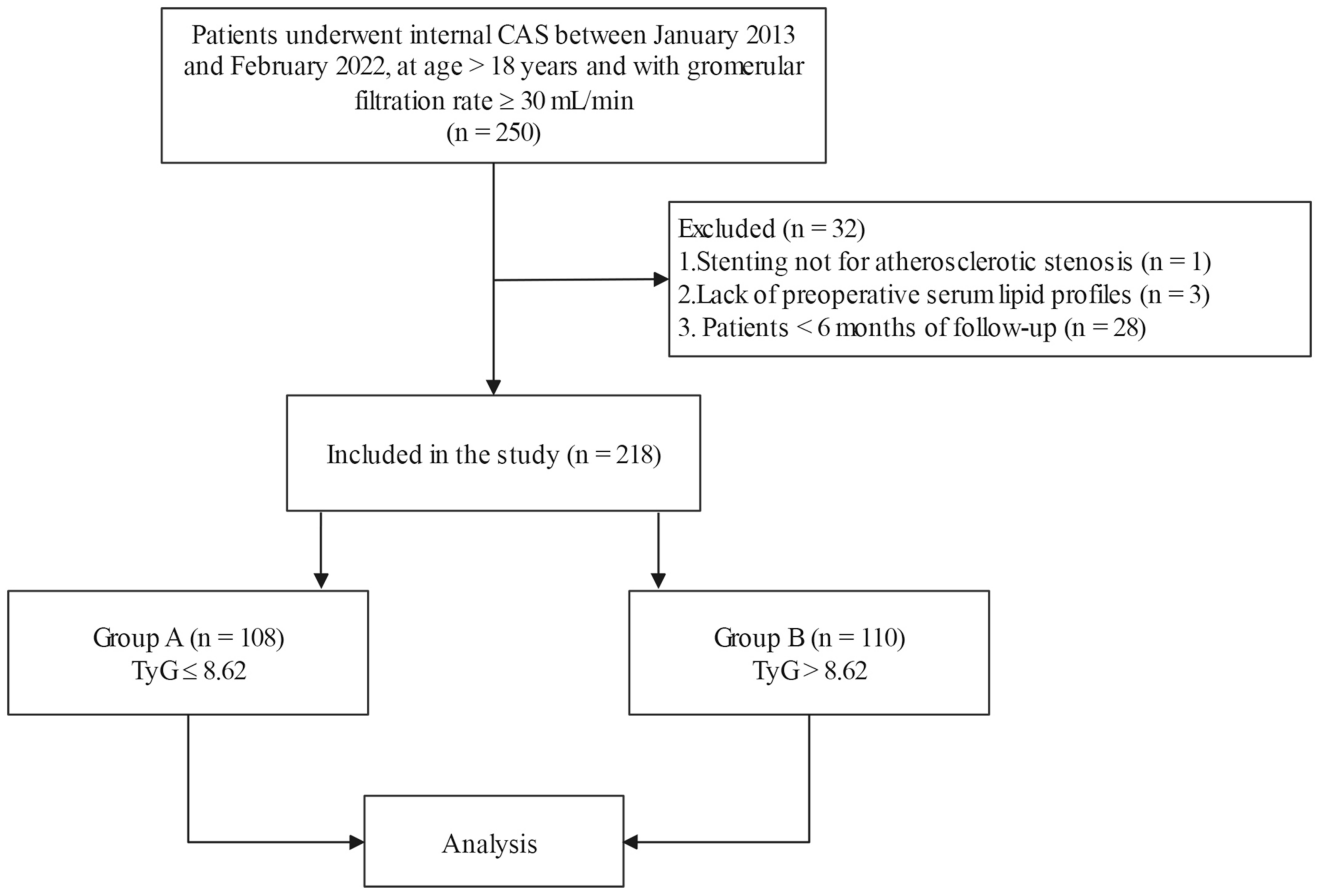
The flowchart of selection. CAS, carotid artery stenting; TyG, triglyceride–glucose.

This was a retrospective cohort study. Eligible participants were patients who underwent internal CAS at Shaoxing People’s Hospital between January 2013 and February 2022, at age > 18 years and with a glomerular filtration rate ≥ 30 ml/min (*n* = 250). Some patients were ruled out due to stenting not for atherosclerotic stenosis (*n* = 1), and lack of preoperative serum lipid profiles (*n* = 3). In addition, considering that a follow-up period that was too short could lead to false negative results, we also removed patients with less than 6 months of follow-up (*n* = 28) (9, 11). The selection flowchart is shown in Figure 1. The date of surgery was considered the start of the follow-up period. The date of the patient’s last visit to our hospital for carotid ultrasound or CTA examination was considered the end of the follow-up period. The endpoint was when ISR was detected by Doppler ultrasound or CTA.

### 2.3. Indication for CAS

The absolute indication for CAS in our hospital was symptomatic stenosis with a stenosis degree ≥ 70% on non-invasive examination or stenosis degree ≥ 50% on digital subtraction angiography (DSA). The relative indications were as follows: (1) asymptomatic stenosis and stenosis degree ≥ 70% on non-invasive examination or stenosis degree ≥ 60% on DSA; (2) asymptomatic stenosis and stenosis degree <70% on non-invasive examination and CTA or other examinations showing unstable plaques; and (3) symptomatic stenosis and a degree of stenosis of 50–60%. All patients provided written informed consent before surgery. A self-expanding stent with an embolic protection device (Abbott Xact Carotid Stent System, Park IL, United States; RX Acculink, Abbott Vascular Solutions, Santa Clara, CA, United States; Protégé, ev3 Inc., Plymouth, MN, United States) was used on all patients. Patients were followed up at 1, 3, and 6 months, and then once a year after CAS.

### 2.4. Data collection and definitions

An experienced neurologist who was not informed of the purpose of the study searched the electronic medical record system, and demographic and clinical data were collected. The collected data included age, sex, body mass index (BMI), blood pressure, current smoking status (yes or no), alcohol consumption (yes or no), symptomatic stenosis (yes or no), medical history (hypertension, dyslipidemia, diabetes mellitus, and coronary heart disease), fasting laboratory data [total cholesterol, TG, high-density lipoprotein cholesterol (HDL-C), low-density lipoprotein cholesterol (LDL-C), FBG, and high-sensitivity C-reactive protein (hs-CRP)], atherosclerotic stenosis (> 50% degree) detected by intraoperative DSA (contralateral ICA, intracranial artery, vertebral artery, subclavian artery), stent type and size, adverse events (transient ischemic attack, ipsilateral stroke, all ischemic strokes, myocardial infarction, and death) during the follow-up, the date of operation, and the date of the last follow-up.

The formula for calculating the TyG index was Ln [fasting triglyceride (mg/dL) × fasting blood glucose (mg/dL)/2] (12), and BMI was calculated as weight/height^2^ (kg/m^2^). Hypertension was defined as a blood pressure ≥ 140/90 mmHg or reception of antihypertensive therapy. Diabetes mellitus was defined as an FBG > 7.0 mmol/l, random blood glucose > 11.1 mmol/l, or reception of hypoglycemic drugs (13). Dyslipidemia was defined as fasting total cholesterol > 6.22 mmol/l, TG ≥ 2.26 mmol/l, LDL-C ≥ 4.14, HDL < 1.04 mmol/l, or reception of lipid-lowering drugs (14). Symptomatic stenosis was defined as neurological dysfunction due to cerebral or retinal ischemia in the ipsilateral carotid region within the previous 6 months (15). ISR was defined as ≥ 50% restenosis inside the stent or involving 5-mm margins. Adverse events were defined as those resulting in readmission during the follow-up period, and included transient ischemic attack, ipsilateral stroke, ischemic stroke, myocardial infarction, and death.

### 2.5. Evaluation of ISR

ISR was evaluated based on the carotid ultrasound or CTA results. Due to the absence of ultrasound velocity criteria for evaluating ISR, an ISR cutoff of 50% or greater was classified as the peak systolic velocity in an ICA greater than 150 cm/s, based on a previous study (16). The North American Symptomatic Carotid Endarterectomy Trial method was applied to determine the degree of restenosis on CTA images. If a patient underwent both Doppler ultrasound and CTA, ISR was considered if either examination met the diagnostic criteria. All images were reevaluated by two experienced radiologists who were unaware of the relevant patient data.

### 2.6. Statistical analysis

Normally or nearly normally distributed data such as age, blood pressure, total cholesterol, TG, HDL-C, and LDL-C are presented as the mean ± standard deviation. The distribution of CRP and the follow-up period was skewed, and therefore expressed as median (interquartile range). Qualitative variables are expressed as numbers (percentages). According to the mean value of the TyG index, the participants were assigned to two groups: group A (TyG index ≤ 8.62) and group B (TyG index > 8.62). A Student’s *t*-test was used for the comparison of normally-distributed parameters between the two groups, the Mann–Whitney *U* test was used for skewed parameters, and the Chi-square test or Fisher’s exact test was used for qualitative variables. We opted for mean imputation for patients with missing BMI values (*n* = 23).

The frequency of restenosis of the two groups was estimated using Kaplan–Meier analysis. The log-rank test was performed to calculate any differences in survival. The Cox regression method was used to analyze the correlation between the continuous TyG index and ISR. The correlation was evaluated by hazard ratio (HR) and 95% confidence interval (CI). Schoenfeld residuals were used to determine the proportional-hazards (PH) assumption. Because the continuous TyG index violated the PH assumption, we added a time interaction term to the model. To select independent variables, candidate variables (*p* < 0.2) in the univariate Cox analysis were included in the backward stepwise regression model. The independent variables (TyG index and minimum stent diameter) identified by the above procedure were then included in the multivariate model, along with variables with potential clinical relevance, including age, sex, symptomatic stenosis, hypertension, diabetes mellitus, dyslipidemia, smoking status, and stent length. We also used restricted cubic splines with four knots at the 5th, 35th, 65th, and 95th centiles to model and visualize the dose–response relationship of the TyG index with the risk of ISR. Subgroup analysis was also performed according to age, sex, BMI, symptomatic stenosis, smoking status, hypertension, and diabetes mellitus. For qualitative data, subgroups were divided according to yes and no. For quantitative data, namely BMI and age, subgroups were divided according to mean values. All statistical analyses were conducted using SAS 9.4 (Cary, NC, United States) software. *p* < 0.05 was considered statistically significant. Statistical power was calculated using PASS 11 software.

## 3. Results

### 3.1. Baseline characteristics

As shown in Table 1, the mean age of the participants was 70.69 years (range: 53–85 years) and 182 (83.5%) participants were males. The prevalence of symptomatic stenosis, hypertension, diabetes mellitus, dyslipidemia, and coronary heart disease was 104 (47.7%), 180 (82.6%), 95 (43.6%), 126 (57.8%), and 27 (12.4%), respectively. In the sample, 92 (42.2%) participants reported consuming alcohol, and 78 (35.8%) were smokers. In terms of stent type, the Abbott Xact Carotid Stent System was used in the majority of participants (87.6%). Based on intraoperative CTA, 69 (31.7%) participants had contralateral ICA stenosis (≥ 50%). The stenosis (≥ 50%) of the intracranial, vertebral, and subclavian arteries were 30 (13.8%), 49 (22.5%), and 12 (5.5%), respectively. Thirty-one participants (14.2%) developed ISR at the end of the study.

**Table 1:** Baseline characteristics of the patients.

Variables	Overall (*n* = 218)	Group A (*n* = 108)	Group B (*n* = 110)	*p*-Value
		TyG ≤ 8.62	TyG **>** 8.62	
Age (year)	70.69 ± 6.84	71.38 ± 7.24	70.01 ± 6.39	0.140
Male, *n* (%)	182 (83.5)	91 (84.3)	91 (82.7)	0.761
BMI (kg/m^2^)	23.39 ± 2.99	23.19 ± 2.55	23.59 ± 3.37	0.322
SBP (mmHg)	148.67 ± 21.42	146.13 ± 21.26	151.17 ± 21.39	0.082
DBP (mmHg)	80.74 ± 11.29	79.93 ± 11.35	81.54 ± 11.22	0.293
Symptomatic, *n*(%)	104 (47.7)	49 (45.4)	55 (50.0)	0.494
Stenostic location, *n*(%)				0.570
Left	93 (42.7)	44 (40.7)	49 (44.5)	
Right	125 (57.3)	64 (59.3)	61 (55.5)	
Current smoking, *n*(%)	92 (42.2)	45 (41.7)	47 (42.7)	0.874
Alcohol consumption, *n*(%)	78 (35.8)	43 (39.8)	35 (31.8)	0.218
*Medical history*				
Hypertension, *n*(%)	180 (82.6)	84 (77.8)	96 (87.3)	0.065
Diabetes mellitus, *n*(%)	95 (43.6)	33 (30.6)	62 (56.4)	< 0.001
Dyslipidemia, *n*(%)	126 (57.8)	50 (46.3)	76 (79.1)	0.001
Coronary heart disease, *n*(%)	27 (12.4)	13 (12.0)	14 (12.7)	0.717
*Laboratory data*				
Total cholesterol (mmol/L)	3.86 ± 0.10	3.56 ± 0.83	4.15 ± 1.07	< 0.001
Triglyceride (mmol/L)	1.37 ± 0.76	0.93 ± 0.26	1.80 ± 0.83	< 0.001
HDL-C (mmol/L)	1.07 ± 0.28	1.14 ± 0.31	1.01 ± 0.25	0.001
LDL-C (mmol/L)	2.35 ± 0.80	2.12 ± 0.71	2.58 ± 0.83	< 0.001
FBG (mmol/L)	5.89 ± 1.98	5.04 ± 1.04	6.72 ± 2.32	< 0.001
hs-CRP (mg/L)	1.52 (0.66, 3.82)	1.65 (0.68, 4.06)	1.44 (0.64, 3.62)	0.864
*Stent-related parameters*				
Type, *n*(%)				0.320
Xact	191 (87.6)	98 (90.7)	93 (84.5)	
PROTÉGÉ	23 (10.6)	8 (7.4)	15 (13.6)	
Acculink	4 (1.8)	2 (1.9)	2 (1.8)	
Minimum diameter (mm), *n*(%)				0.790
6	84 (38.5)	42 (38.9)	42 (38.2)	
7	108 (49.5)	51 (47.2)	57 (51.8)	
8	23 (40.6)	13 (12.0)	10 (9.1)	
9	3 (1.4)	2 (1.9)	1 (0.9)	
Length (mm), *n*(%)				0.193
30	80 (36.7)	35 (32.4)	45 (40.9)	
40	138 (63.3)	73 (67.6)	65 (59.1)	
*Atherosclerotic stenosis*				
Contralateral ICA, *n* (%)	69 (31.7)	30 (27.8)	39 (35.5)	0.223
Intracranial artery, *n*(%)	30 (13.8)	16 (14.8)	14 (12.7)	0.655
Vertebral artery, *n*(%)	49 (22.5)	22 (20.4)	27 (24.5)	0.460
Subclavian artery, *n*(%)	12 (5.5)	6 (5.6)	6 (5.4)	0.974

TyG: Triglyceride-glucose; BMI: body mass index; SBP: systolic blood pressure; DBP: diastolic blood pressure; HDL-C: high-density lipoprotein cholesterol; LDL-C: high-density lipoprotein cholesterol; FBG: fasting blood glucose; hs-CRP: high sensitivity C-reactive protein; ICA: internal carotid artery.

The participants were assigned to two groups according to the mean TyG index value (*M* = 8.62): 108 to group A (TyG index ≤ 8.62) and 210 to group B (TyG index > 8.62). Diabetes mellitus (56.4 vs. 30.6%, *p* < 0.001) and dyslipidemia (79.1 vs. 46.3%, *p* = 0.001) were more prevalent in group B. Within the laboratory data, total cholesterol (4.15 ± 1.07 vs. 3.56 ± 0.83 mmol/l, *p* < 0.001), TG (1.80 ± 0.83 vs. 0.93 ± 0.26 mmol/l, *p* < 0.001), LDL-C (2.58 ± 0.83 vs. 2.12 ± 0.71 mmol/l, *p* < 0.001), and FBG (6.72 ± 2.32 vs. 5.04 ± 1.04 mmol/l, *p* < 0.001) levels were higher in group B, but HDL-C (1.01 ± 0.25 vs. 1.14 ± 0.31 mmol/l, *p* = 0.001) levels were lower. However, no difference was observed in hs-CRP levels. Other baseline variables, including age, sex, BMI, symptomatic stenosis, prevalence of current smoking and hypertension, and stent-related parameters were not statistically different. Meanwhile, we observed no significant differences in stenosis of the contralateral ICA, intracranial artery, vertebral artery, or subclavian artery.

### 3.2. Tests on PH assumption

**Figure 2:**
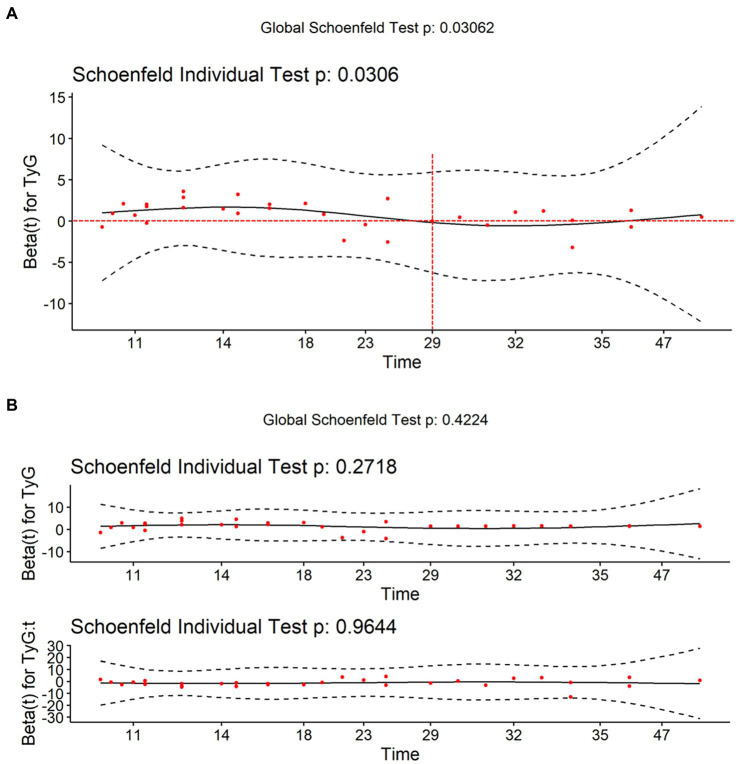
Visualization of Schoenfeld residuals. The red points represent the Schoenfeld residuals. The black curve represents the smooth curve of Schoenfeld residuals against time. The black dashed line represents the confidence interval of the smooth curve. The red dashed line represents zero horizontal line. **(A)** The smooth curve fluctuates up and down the zero horizontal line over time, which does not meet the PH assumption. **(B)** After adding 29 months as the time interaction term, the smooth curve lies on the zero horizontal line, satisfying the proportional-hazards assumption (*p* > 0.05).

According to the tests on Schoenfeld residuals, the continuous TyG index violated the PH assumption (*p* = 0.031). If the PH assumption holds, the change in Schoenfeld residuals over time should fluctuate randomly above and below the zero horizontal line in the Schoenfeld residual plot. However, sometimes it is difficult to evaluate the trend of scatter. We can use the local-weighted scatterplot smoothing function to plot the smooth curve of the Schoenfeld residual, which helps us to make judgments. Theoretically, under the null assumption of PHs, this function would have a slope of zero (17). Figure 2A shows that the curve fluctuates up and down with time, which does not meet the PH assumption. Therefore, we added a time interaction term to the model. In this study, combined the extreme and zero points of the curve with the results of model construction (optimally satisfied the PH assumption), we finally chose 29 months as the proper cutoff time point (18). Figure 2B shows that after adding 29 months as the time interaction term, the smooth curve lies on the zero horizontal line, satisfying the PH assumption (*p* > 0.05). The tests on Schoenfeld residuals of all variables and the entire model are presented in Supplementary Table S1. All variables and the entire model were in accordance with the PH assumption after including the time interaction term (*p* > 0.05).

### 3.3. The relevance of the TyG index on the risk of restenosis

**Figure 3:**
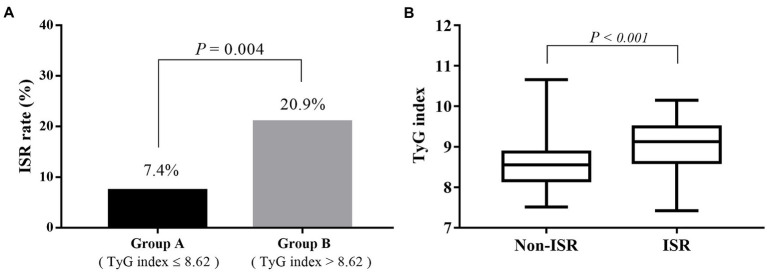
The relevance of the preoperative TyG index on the risk of restenosis. **(A)** The restenosis rate of participants in group B was significantly higher than that in group A (20.9 vs. 7.4%, *p* = 0.004). **(B)** The TyG index was significantly higher in the restenosis group than in the non-restenosis group (8.55 ± 0.53 vs. 9.02 ± 0.65, *p* < 0.001). TyG, triglyceride–glucose.

**Figure 4:**
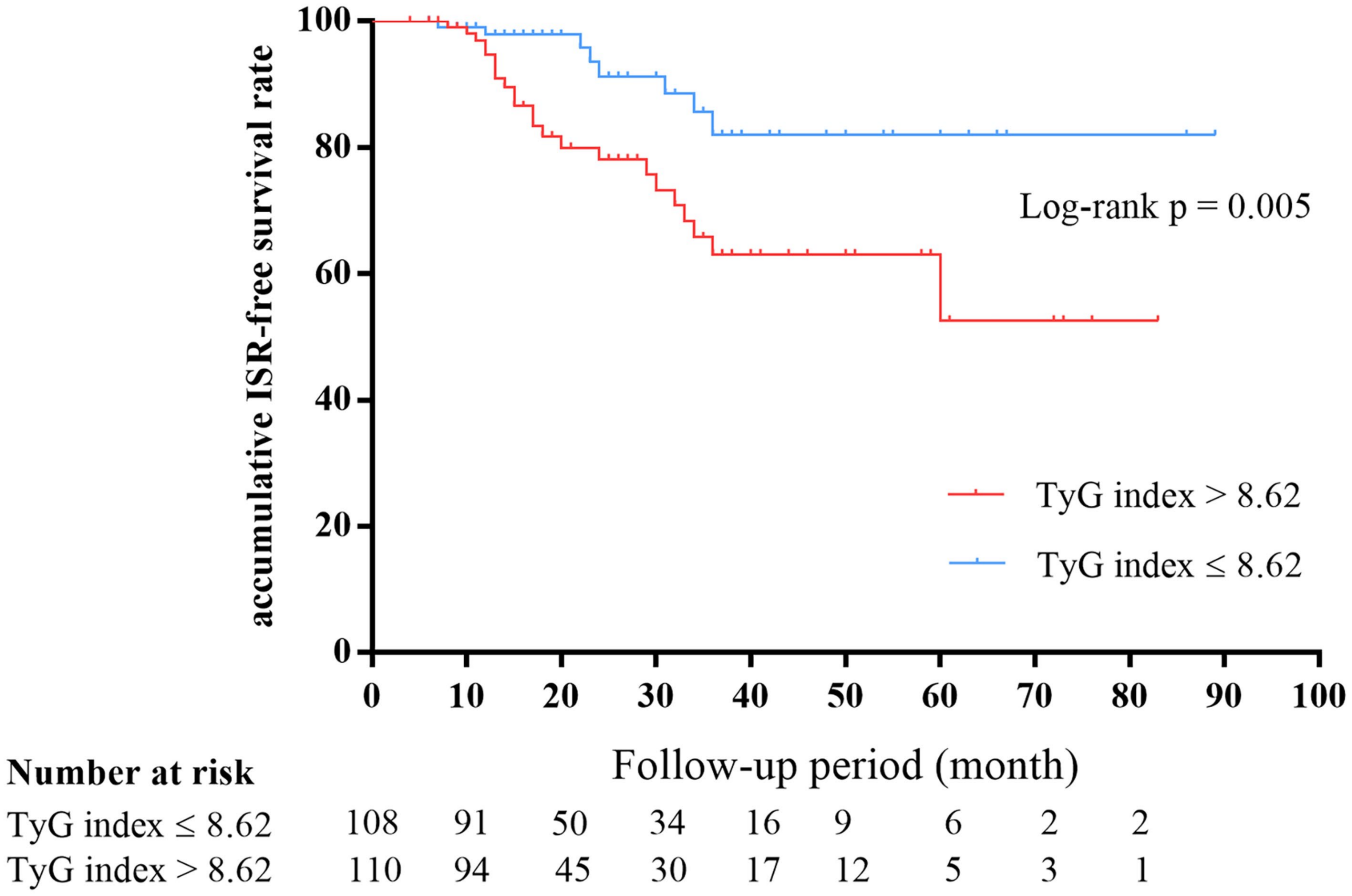
Kaplan–Meier survival estimates showed that the cumulative restenosis-free rate in the high TyG index group was significantly lower than that in the low group (*p* = 0.005). TyG, triglyceride–glucose.

The number of participants with restenosis was 23 (20.9%) in group B and eight (7.4%) in group A, and this difference was significant (*p* = 0.004; Figure 3A). Meanwhile, the TyG index was significantly higher in the restenosis group than in the non-restenosis group (8.55 ± 0.53 vs. 9.02 ± 0.65, *p* < 0.001; Figure 3B). The 12- and 24-month cumulative restenosis-free rates in group A were 99.0 and 91.2%, respectively versus 94.7 and 78.3%, respectively in group B. The log-rank test showed that the cumulative restenosis-free rate in group B was significantly lower than that in group A (*p* = 0.005; Figure 4).

**Figure 5:**
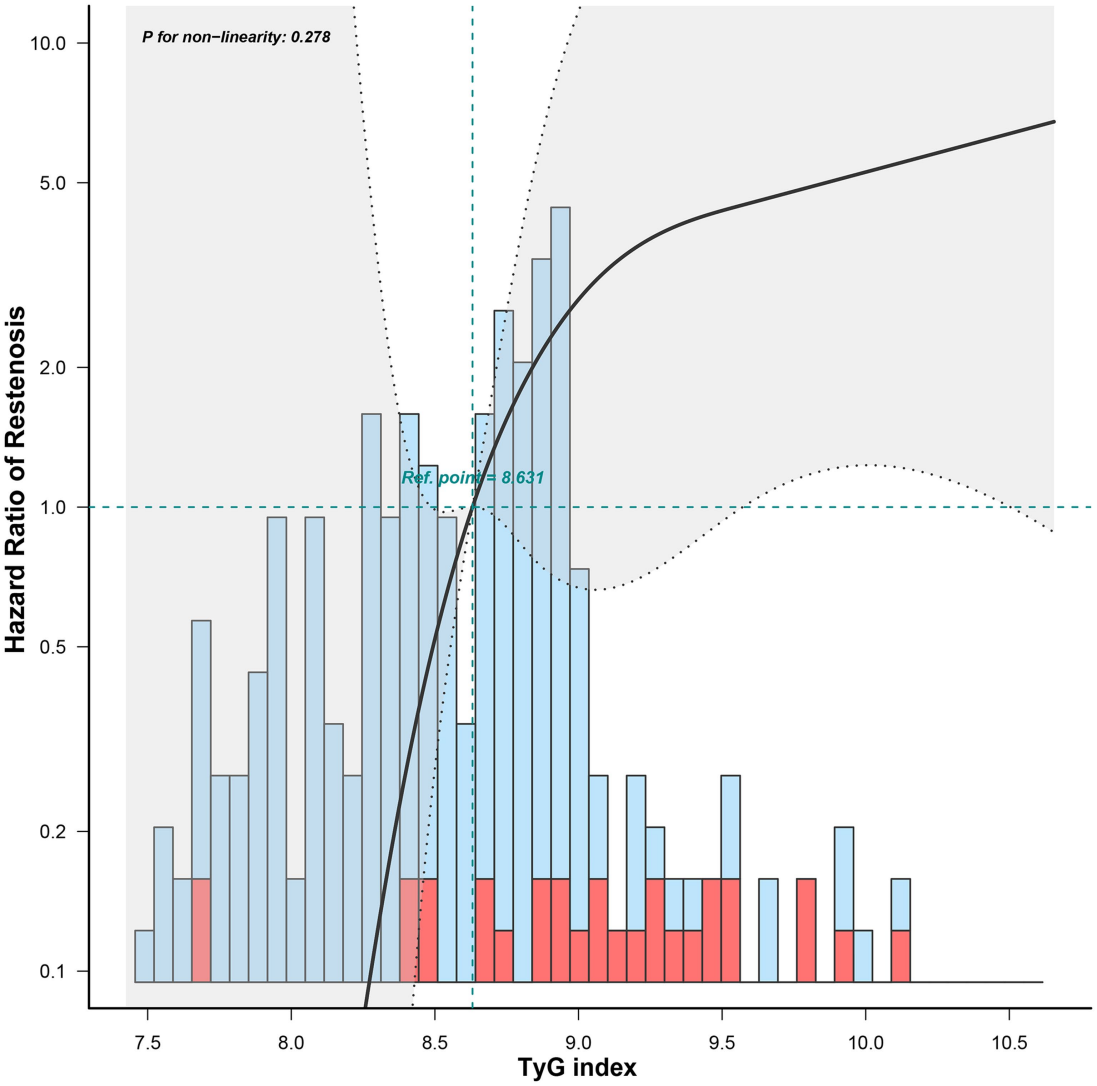
The dose–response relationship of the preoperative TyG index with restenosis.

The univariate Cox regression analysis results are presented in Table 2. The TyG index (*p* < 0.001), BMI (*p* = 0.037), TG (*p* = 0.002), FBG (*p* = 0.007), and rate of diabetes mellitus (*p* = 0.012) were significantly different between the groups. Variables with *p* < 0.2 were entered into the backward stepwise regression model. TyG index and minimum stent diameter were entered as independent predictors of ISR, as shown in Table 3. At < 29 months of follow-up, the risk of restenosis increased by 5.135 (2.463–10.709) times for every 1-unit increase in the TyG index. Participants with a minimum stent diameter of ≥ 7 mm were 0.463 (0.221–0.969) times more likely to develop restenosis than those with a minimum stent diameter of 6 mm. After further adjustment for potential clinical relevance, the TyG index (4.347, 1.886–10.023) was still a robust predictor of ISR. Figure 5 shows the dose–response relationship of the TyG index with ISR. There was a trend towards an increased risk of restenosis with higher TyG indexes.

**Table 2:** Univariate Cox regression.

Variables	Grade	*HR (95%CI)*	*P*
TyG*	< 29 months	4.331(2.219–8.451)	< 0.001
	≥ 29 months	1.348(0.464–3.914)	0.583
Age (year)		0.999(0.950–1.052)	0.981
Sex	Female vs. Male	1.807(0.807–4.047)	0.151
BMI		1.175(1.009–1.367)	0.037
Symptomatic	Yes vs. No	0.869(0.425–1.773)	0.699
Stenostic location	Left vs. Right	1.164(0.570–2.377)	0.677
Total cholesterol (mmol/L)		1.328(0.959–1.839)	0.087
TG (mmol/L)		1.606(1.189–2.169)	0.002
HDL-C (mmol/L)		0.474(0.129–1.751)	0.263
LDL-C (mmol/L)		1.369(0.922–2.033)	0.119
FBG (mmol/L)		1.176(1.045–1.323)	0.007
hs-CRP (mg/L)		0.984(0.948–1.021)	0.386
Current smoking	Yes vs. No	0.771(0.374–1.589)	0.48
Alcohol consumption	Yes vs. No	0.689(0.317–1.497)	0.347
*Medical history*			
Hypertension	Yes vs. No	0.574(0.256–1.290)	0.179
Coronary heart disease	Yes vs. No	0.414(0.056–3.062)	0.388
Dyslipidemia	Yes vs. No	1.577(0.742–3.353)	0.236
Diabetes mellitus	Yes vs. No	2.568(1.230–5.361)	0.012
*Stenosis (≥ 50%)*			
Contralateral ICA	Yes vs. No	1.085(0.511–2.305)	0.832
Intracranial artery	Yes vs. No	1.042(0.364–2.983)	0.938
Vertebral artery	Yes vs. No	1.359(0.607–3.043)	0.456
Subclavian artery	Yes vs. No	1.206(0.288–5.058)	0.798
*Stent-related parameters*			
Type	PROTG and Acculink vs. Xact	0.851(0.258–2.805)	0.791
Minimum diameter	7 mm & above vs. 6 mm	0.570(0.282–1.155)	0.119
Length	40 mm vs. 30 mm	1.525(0.700–3.320)	0.288

* Calculate the HR of different time periods based on a time interaction term.

TyG: Triglyceride-glucose; BMI: body mass index; HDL-C: high-density lipoprotein cholesterol; LDL-C: high-density lipoprotein cholesterol; FBG: fasting blood glucose; hs-CRP: high sensitivity C-reactive protein; ICA: internal carotid artery.

**Table 3:** Multivariate Cox regression.

Variables	Grade	HR(95%CI)	*p*
*Backward stepwise regression*			
TyG	< 29 months	5.135 (2.463–10.709)	<0.001
	≥ 29 months	1.410 (0.492–4.040)	0.523
Minimum diameter of stent	7 mm and above vs. 6 mm	0.463 (0.221–0.969)	0.041
*Multivariate adjustment*			
TyG	< 29 months	4.347 (1.886–10.023)	0.001
	≥ 29 months	1.009 (0.310–3.280)	0.988
Age		1.046 (0.980–1.117)	0.18
Sex	Female vs. Male	1.460 (0.574–3.715)	0.427
Symptomatic	Yes vs. No	1.164 (0.522–2.594)	0.711
Hypertension	Yes vs. No	0.609 (0.245–1.512)	0.285
Dyslipidemia	Yes vs. No	1.217 (0.506–2.930)	0.661
diabetes mellitus	Yes vs. No	2.007 (0.847–4.755)	0.114
Current smoking	Yes vs. No	0.623 (0.262–1.482)	0.285
Minimum diameter of stent	7 mm and above vs. 6 mm	0.377 (0.169–0.842)	0.017
Length of stent	40 mm vs. 30 mm	1.071 (0.441–2.602)	0.879

TyG:Triglyceride-glucose.

**Table 4:** Adverse events and follow-up period of the patients.

Variables	Overall (*n* = 218)	Group A (*n* = 108)	Group B (*n* = 110)	*p*-Value
		TyG ≤ 8.62	TyG > 8.62	
*Adverse events, n(%)*				
total	27 (12.4)	8 (7.4)	19 (17.3)	0.027
transient ischemic attack	1 (0.5)	0 (0.0)	1 (0.9)	1.000
ipsilateral stroke	5 (2.3)	2 (1.9)	3 (2.7)	0.633
all ischemic stroke	15 (6.9)	5 (4.6)	10 (9.1)	0.193
myocardial infarction	5 (2.3)	1 (0.9)	4 (3.6)	0.377
death	1 (0.5)	0 (0.0)	1 (0.9)	1.000
*Follow-up period (month)*	17.00 (12.00, 36.00)	18.00 (13.0, 34.00)	15.50 (12.00, 33.25)	0.616

### 3.4. Subgroup analysis

**Figure 6:**
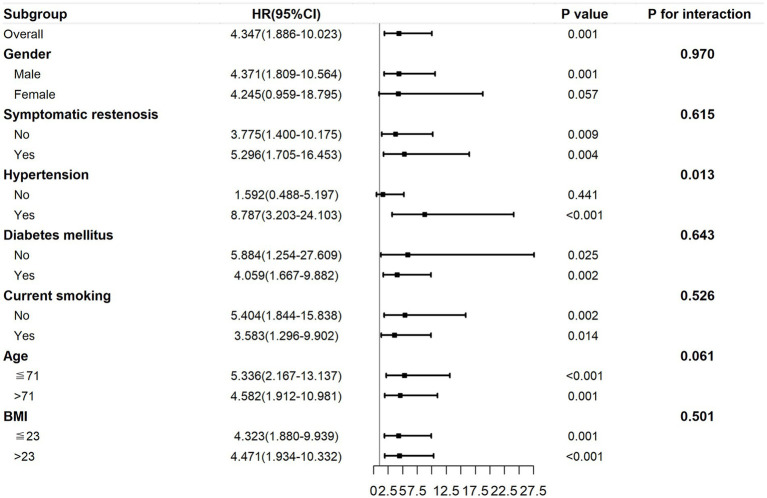
The subgroup analysis of the association between the preoperative TyG index and ISR within 29 months post-surgery. The TyG index was adjusted for variables with *p* < 0.2 in univariate Cox regression and variables with potential clinical relevance. TyG, triglyceride–glucose; ISR, in-stent restenosis.

The subgroup analysis within 29 months post-surgery is presented in Figure 6. The association between the TyG index and ISR was still consistent (*P* for interactions >0.05) in the subgroups of sex (male or female), BMI (≤ 23 or > 23 kg/m^2^), symptomatic stenosis (yes or no), current smoking (yes or no), and diabetes mellitus (yes or no). However, there was a significant difference in the hypertension subgroups (yes or no; *p* for interactions = 0.013) and a marginally significant difference in the age subgroups (≤ 71 or > 71 years; *p* = 0.061). HRs tended to be higher in the age ≤ 71 years subgroup (*p* < 0.001) and participants with hypertension (*p* < 0.001).

### 3.5. Adverse events

During the follow-up period, adverse events occurred in 27 (12.4%) participants. The prevalence of transient ischemic attack, ipsilateral stroke, all ischemic stroke, myocardial infarction, and death were one (0.5%), five (2.3%), 15 (6.9%), five (2.3%), and one (0.5%), respectively. The participants in group B had a higher incidence of total adverse events (17.3% vs. 7.4%, *p* = 0.027). In addition, the median follow-up period was 17 months (range: 6–89 months). The follow-up period was not significantly different in the two groups[18.00 (13.0, 34.00) vs. 15.50 (12.00, 33.25) months, *p* = 0.616] (Table 4).

### 3.6. Statistical power

A Cox regression of the log hazard ratio on a covariate with a standard deviation of 0.5690 based on a sample of 220 observations achieves 90.3% power at a 0.05 significance level to detect a regression coefficient equal to 1.1560. The sample size was adjusted since a multiple regression of the variable of interest on the other covariates in the Cox regression is expected to have an *R*-squared of 0.215. The sample size was adjusted for an anticipated event rate of 0.142. The results showed that the statistical power of the sample size of 218 was sufficient. A detailed report of the statistical power calculations is provided in the Supplementary material.

## 4. Discussion

The Cox regression model is most commonly used for time-to-event analyses. However, it has to satisfy the PH assumption that the HRs in the two groups remain constant over time. In our study, we demonstrated that the preoperative TyG index violated the PH assumption, implying that it had a time-varying effect on ISR. Within 29 months post-surgery, an increasing preoperative TyG index was linked to a significant increased risk of restenosis. However, after 29 months, the effect was decreased, although not statistically significant. This may imply that we should be alert to the possibility of short-term restenosis in patients with a higher preoperative TyG index. The hazard reduction over time may be due to the instability of the TyG index during long-term follow-up. The TyG index can be influenced by medication and disease progression, thus leading to a weakening of the association. In addition, inadequate sample size may also have contributed to this result. To further clarify the relationship between TyG index and restenosis, restricted cubic spline was also used. Restricted cubic spline is one of the commonly used methods to study nonlinear relationships. This method can fit the curve relationship of independent variables and realize the association analysis between continuous exposure and outcome (19, 20). In this study, the risk of restenosis increased with the increase in the TyG index. Moreover, we stratified baseline variables and demonstrated that a higher TyG index posed greater risks to younger patients or those with hypertension.

There are some new studies about the relationship between the TyG index and carotid atherosclerosis. Wang et al. (21) determined that a higher TyG index was linked to the occurrence of carotid plaques in non-diabetic participants through a large sample study of 24,895 urban workers. Li et al. (22) discovered that the TyG index and carotid plaques were significantly associated in participants with coronary heart disease. Li et al. (23) investigated the relationship between the TyG index and carotid atherosclerosis in a general population aged ≥ 40 years from five cities and discovered that the TyG index had a significant relationship with the progression of carotid intima-media thickness, plaques, and stenosis. These findings indirectly support our research finding that the TyG index may contribute to restenosis after CAS for atherosclerotic carotid stenosis. Additionally, the TyG index has been confirmed to be related to hypertension (24) and diabetes mellitus (25, 26), which are recognized as risk factors for ISR. However, the relationship between TyG index and ISR has rarely been studied. Zhu et al. (9) retrospectively enrolled participants who underwent percutaneous coronary stenting. They discovered that a higher TyG index increased the risk of ISR through multivariate logistic regression. Since the pathogenesis of restenosis is similar between coronary and carotid stents (27, 28), we hypothesized that the TyG index might also contribute to ISR after CAS. In contrast to the aforementioned research, the participants were followed up over a much longer period of time (6–89 months) than were those in the Zhu et al. study (6–24 months). Moreover, we used survival analysis and multivariate Cox regression and demonstrated that the effect of preoperative TyG index on ISR varied with time. Notably, during the follow-up, we also observed a higher prevalence of adverse events in participants with a high TyG index. This result is in line with the previous findings. In patients with acute coronary syndrome, for instance, a higher TyG index can increase the prevalence of myocardial infarction, stroke, and other cardiovascular events (29, 30).

The mechanism underlying the positive correlation between the TyG index and ISR remains unclear. We suspected that this might be related to pathophysiological changes caused by IR. First, it has been established that neointimal hyperplasia is the primary cause of ISR in its early stages (within 2 years after stenting) (31). IR can induce proliferation and migration of vascular smooth muscle cells, leading to endointimal hyperplasia (32). Second, long-term restenosis after stenting may be associated with recurrent atherosclerosis and plaque formation (33, 34). IR can lead to endothelial dysfunction by reducing nitric oxide production and increasing the release of procoagulant factors, thus leading to recurrent atherosclerosis (35, 36). Previous studies have reported that some perioperative acute-phase reactants such as hs-CRP (16) and leukocytes (37) are related to ISR. Short-term elevation of these inflammatory indicators is often associated with endothelial injury caused by stent implantation. However, the development of ISR is a long-term process. Unlike these inflammatory indicators, the TyG index has a persistent effect on the human body.

It is well known that advanced age, female sex, hypertension, diabetes mellitus, smoking, and symptomatic stenosis are positively associated with restenosis after CAS (38–40). Interestingly, in our subgroup analysis, younger patients were at a higher risk of TyG-related restenosis. The reason for this phenomenon remains unclear. The results of our study indicate that more attention should be paid to the control of the TyG index in young patients. Our result was consistent with that of Li et al. (23), in which middle-aged participants had a higher prevalence of intima-media thickness and plaque formation than elderly participants. Li et al. (22) also reported that the correlation between the TyG index and carotid atherosclerosis was stronger in middle-aged participants. However, findings reported by Zhu et al. (9) were contrary to our findings. In their study, the association between the TyG index and ISR after coronary stenting was higher in participants older than 65 years. The reason for this may lie in the different study populations.

This study had some limitations. First, it was a retrospective cohort study. As a result, the follow-up intervals were not strictly set, which could have led to inaccurate endpoint times in some participants. Second, the sample size was small, particularly with respect to the number of participants with ISR. Although this is partly due to the low incidence rate of ISR, a prospective study with a larger sample size could provide more evidence. Third, Doppler ultrasound and CTA were used to assess ISR in our study. However, ultrasound velocity criteria for evaluating ISR are lacking. This is one of the reasons why restenosis rates vary widely among studies. Fourth, the TyG index was obtained only from preoperative laboratory data, whereas serum TG and FBG levels are dynamic parameters that can be influenced by medication and disease progression. Therefore, the association between the trajectory of the TyG index in follow-up and the risk of ISR needs further study.

In conclusion, the preoperative TyG index had a positive effect on the risk of short-term restenosis after CAS within 29 months post-surgery. As a simple and low-cost index, the TyG index may be employed to stratify patients based on their risk of restenosis after carotid artery stenting.

## Data availability statement

The raw data supporting the conclusions of this article will be made available by the authors, without undue reservation.

## Ethics statement

The studies involving human participants were reviewed and approved by Academic Ethics Committee of Shaoxing People’s Hospital. Written informed consent for participation was not required for this study in accordance with the national legislation and the institutional requirements.

## Author contributions

BW and S-sZ were responsible for collecting the data of the patients. X-tL and J-bZ contributed to the study design. S-sZ wrote the manuscript. J-bZ and S-sZ performed the data analysis. All authors contributed to the article and approved the submitted version.

## Funding

This study was supported by the General Project of Zhejiang Health Science, Technology Plan (Nos. 2021KY1147 and 2023KY1248), Shaoxing People’s Hospital Youth Research Fund (2022YB06), and Shaoxing Medical Key Discipline (2019SZD05).

## Conflict of interest

The authors declare that the research was conducted in the absence of any commercial or financial relationships that could be construed as a potential conflict of interest.

## Publisher’s note

All claims expressed in this article are solely those of the authors and do not necessarily represent those of their affiliated organizations, or those of the publisher, the editors and the reviewers. Any product that may be evaluated in this article, or claim that may be made by its manufacturer, is not guaranteed or endorsed by the publisher.
