# Supplementary material for: The preoperative triglyceride-glucose index has a positive effect on predicting the risk of short-term restenosis after carotid artery stenting: a retrospective cohort study
Source: Front Neurol. 2023 Apr 17;14:1159601. doi: 10.3389/fneur.2023.1159601 (PMC10149666; doi:10.3389/fneur.2023.1159601)
Supplement: Supplementary file 1 [file Table_1.pdf]

Table S1. Tests of Schoenfeld's residuals

Model	Variables	x^2	*P*
Univariate	TyG	4.674	0.031
	GLOBAL	4.674	0.031
Univariate	TyG	1.207	0.272
+ Time interaction term	Time interaction term	0.002	0.964
	GLOBAL	1.724	0.422
Backward stepwise regression	TyG	1.17	0.279
+ Time interaction term	Minimum diameter	0.712	0.399
	Time interaction term	0.011	0.917
	GLOBAL	2.877	0.411
Multivariate Adjustment	TyG	1.76	0.185
+ Time interaction term	age	0.03	0.862
	gender	3.247	0.072
	Symptomatic	0.13	0.718
	Hypertension	1.599	0.206
	Dyslipidemia	0.176	0.675
	diabetes mellitus	1.475	0.225
	Current smoking	1.503	0.22
	Minimum diameter	0.776	0.378
	Length	0.442	0.506
	Time interaction term	0.051	0.821
	GLOBAL	12.076	0.358

TyG：Triglyceride-glucose
