# Supplementary material for: The preoperative triglyceride-glucose index has a positive effect on predicting the risk of short-term restenosis after carotid artery stenting: a retrospective cohort study
Source: Front Neurol. 2023 Apr 17;14:1159601. doi: 10.3389/fneur.2023.1159601 (PMC10149666; doi:10.3389/fneur.2023.1159601)

Cox Regression Power Analysis

Numeric Results

Power	Sample Size (N)	Reg. Coef. (B)	S.D. of X1 (SD)	Event Rate (P)	R-Squared X1 vs Other X's (R2)	Two-Sided Alpha	Beta
0.16408	20	1.1560	0.5690	0.1420	0.2150	0.05000	0.83592
0.45122	70	1.1560	0.5690	0.1420	0.2150	0.05000	0.54878
0.67210	120	1.1560	0.5690	0.1420	0.2150	0.05000	0.32790
0.81684	170	1.1560	0.5690	0.1420	0.2150	0.05000	0.18316
0.90275	220	1.1560	0.5690	0.1420	0.2150	0.05000	0.09725
0.95038	270	1.1560	0.5690	0.1420	0.2150	0.05000	0.04962

References

Hsieh, F.Y. and Lavori, P.W. 2000. 'Sample-Size Calculations for the Cox Proportional Hazards Regression Model with Nonbinary Covariates', Controlled Clinical Trials, Volume 21, pages 552-560.

Schoenfeld, David A. 1983. 'Sample-Size Formula for the Proportional-Hazards Regression Model', Biometrics, Volume 39, pages 499-503.

Report Definitions

Power is the probability of rejecting a false null hypothesis. It should be close to one.
N is the size of the sample drawn from the population.
B is the size of the regression coefficent to be detected
SD is the standard deviation of X1.
P is the event rate.
R2 is the R-squared achieved when X1 is regressed on the other covariates.
Alpha is the probability of rejecting a true null hypothesis.
Beta is the probability of accepting a false null hypothesis.

Summary Statements

A Cox regression of the log hazard ratio on a covariate with a standard deviation of 0.5690 based on a sample of 20 observations achieves 16% power at a 0.05000 significance level to detect a regression coefficient equal to 1.1560. The sample size was adjusted since a multiple regression of the variable of interest on the other covariates in the Cox regression is expected to have an R-Squared of 0.2150. The sample size was adjusted for an anticipated event rate of 0.1420.

Cox Regression Power Analysis

Chart Section

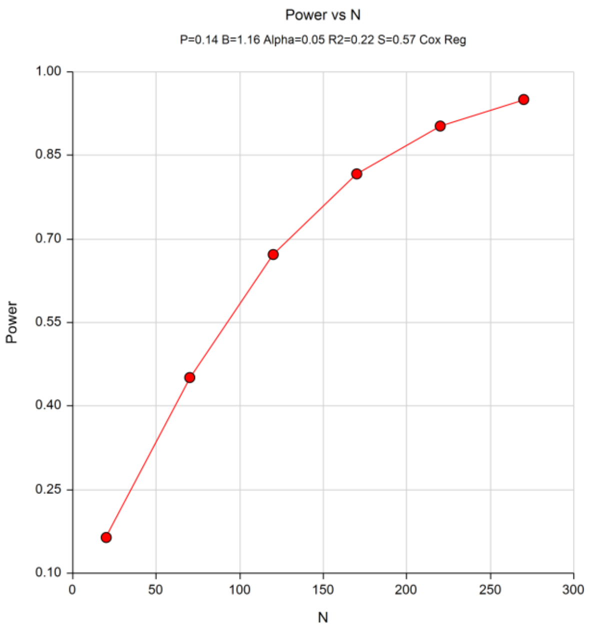

Supplement: Supplementary file 2 [file Data_Sheet_1.pdf]
